# A rare case of pure non-gestational ovarian choriocarcinoma: Diagnostic mimicry and management strategies

**DOI:** 10.18632/oncoscience.622

**Published:** 2025-07-28

**Authors:** Naina Kumar, Abhishek Arora, Gunvanti Rathod, Mishu Mangla, Aparna Setty, Pooja T. Rathod, Banka Sai Swetha

**Affiliations:** ^1^Department of Obstetrics and Gynaecology, All India Institute of Medical Sciences, Bibinagar 508126, Hyderabad, Telangana, India; ^2^Department of Radiodiagnosis, All India Institute of Medical Sciences, Bibinagar 508126, Hyderabad, Telangana, India; ^3^Department of Pathology, All India Institute of Medical Sciences, Bibinagar 508126, Hyderabad, Telangana, India

**Keywords:** chemotherapy, ectopic pregnancy, germ cell tumor, gestational ovarian choriocarcinoma, non-gestational ovarian choriocarcinoma

## Abstract

Background: Non-gestational ovarian choriocarcinomas (NGOC) are rare, distinct, highly aggressive tumors, primarily affecting young women. It accounts for less than 0.6% of malignant ovarian germ cell tumors. It is associated with a poorer prognosis compared to gestational choriocarcinoma.

Case Presentation: A 36-year-old woman (P2L2) presented with intermittent heavy menstrual bleeding for the past three months. The urinary pregnancy test was positive. On abdominal examination, a solid mass consistent with 20-weeks gravid uterus was palpated in right iliac fossa. Bimanual pelvic examination revealed uterus deviated to the left and large (~12 × 10 cm) predominantly solid mass arising from right adnexa, adherent to the uterus. A mobile cystic mass (6 × 5 cm) was palpated in the left fornix. Ultrasonography showed normal-sized uterus with no gestational sac and a well-defined, solid-cystic right adnexal mass (10.2 × 7.8 × 7.8 cm) with vascularized solid areas and hemorrhage, initially suggesting an ectopic pregnancy. Serum β-hCG was markedly elevated (262,809 mIU/mL; normal level <5.0 mIU/mL). Magnetic Resonance Imaging (MRI) and Contrast-enhanced Computed Tomography (CECT) revealed right ovarian germ cell tumor, likely choriocarcinoma, without evidence of metastatic disease. On staging laparotomy, hemorrhagic right tubo-ovarian mass (8.5 × 8 × 7 cm) and left ovarian serous cystadenoma (8 × 7 × 3.5 cm) were identified. Histopathology and genomic studies confirmed stage IA1 NGOC. Patient completed two cycles of adjuvant chemotherapy with Bleomycin, Etoposide, Cisplatin, achieving complete response (β-hCG <5 mIU/mL), and is following up with serial β-hCG monitoring and CT scans for two years.

Conclusions: NGOC closely mimics ectopic pregnancy and gestational trophoblastic disease and requires early diagnosis with prompt surgical and chemotherapeutic intervention to optimize outcomes.

## INTRODUCTION

Ovarian choriocarcinoma is an exceptionally rare type of ovarian malignancy, broadly categorized into two distinct forms: gestational ovarian choriocarcinoma (GOC) and non-gestational ovarian choriocarcinoma (NGOC) [1–3]. NGOC can be further classified into two subtypes—pure, consisting solely of choriocarcinomatous tissue, and mixed, which includes other germ cell tumor elements like immature teratomas, endodermal sinus tumors, embryonal carcinomas, and dysgerminomas [1]. GOC, a subtype of gestational choriocarcinoma, is typically associated with prior pregnancies and often presents alongside a well-developed corpus luteum. The estimated annual global incidence of GOC is approximately 1 in 369 million [1]. In contrast, NGOC is unrelated to pregnancy and accounts for less than 0.6% of all malignant ovarian germ cell tumors [1, 3, 4–6]. Isolated NGOC is an exceptionally rare neoplasm, composed entirely of choriocarcinomatous elements, and is associated with a poorer prognosis compared to its gestational counterpart (GOC) [2, 7].

NGOC primarily affects young, reproductive-aged women and frequently presents with metastatic disease at the time of diagnosis [8]. Non-gestational choriocarcinoma, particularly of ovarian origin, can closely mimic ectopic pregnancy due to overlapping clinical features and elevated β-hCG levels. Both conditions commonly present with vaginal bleeding, abdominal pain, adnexal masses, and a positive pregnancy test, making initial differentiation challenging [2, 9, 10]. In pediatric cases, NGOC may also present with precocious puberty [1]. Most reported cases involve unilateral ovarian masses, although rare instances of bilateral NGOC have been described [11]. Due to the tumors’ usual unilateral nature and their clinical resemblance to ectopic pregnancy, initial misdiagnosis is common [10, 12].

NGOCs are highly vascular tumors due to the invasive nature of trophoblastic cells, often presenting with bleeding that may necessitate significant blood transfusions or activation of massive transfusion protocols [8, 10, 13].

Historically, the diagnosis of NGOC was primarily based on clinical history, particularly the absence of recent sexual activity or antecedent pregnancy, which made NGOC more likely in such cases [12, 14]. However, advances in genetic analysis now allow for more definitive differentiation between gestational and non-gestational choriocarcinomas. Gestational choriocarcinoma originates from a pregnancy and thus contains both maternal and paternal genetic material. In contrast, non-gestational choriocarcinoma arises independently of pregnancy and contains only maternal DNA. Therefore, the detection of paternal DNA confirms a diagnosis of GOC, whereas its absence supports a diagnosis of NGOC [1]. Fluorescence in situ hybridization (FISH), using probes targeting X and Y chromosome centromeres, has emerged as a reliable screening method to distinguish between these two entities [15]. Currently, no specific immunohistochemical (IHC) markers exist to differentiate GOC from NGOC [3]; however, making this distinction remains crucial, as the two forms differ significantly in prognosis and require distinct therapeutic strategies [3, 10].

This case report describes a 36-year-old woman who presented with a three-month history of intermittent heavy menstrual bleeding and a positive urinary pregnancy test. Initial ultrasound suggested an ectopic pregnancy. However, further evaluation with Magnetic resonance imaging (MRI) and contrast-enhanced computed tomography (CECT) revealed a right ovarian germ cell tumor, most likely ovarian choriocarcinoma, without evidence of distant metastasis. The patient underwent surgical management followed by adjuvant chemotherapy. Histopathology, IHC, and genomic studies confirmed the diagnosis of stage IA1 pure NGOC of the right ovary.

## CASE REPORT

A 36-year-old woman, para 2 live 2 (P2L2), with a history of two caesarean sections (the first for placenta previa and the second for previous caesarean section), last delivering six years ago, presented to the gynecology outpatient department with complaints of intermittent heavy menstrual bleeding for the past three months. She reported a history of using combined oral contraceptive pills for 10 days, four months ago, to delay her periods. Her previous menstrual cycles were regular, with bleeding lasting 4–5 days every 28–30 days. She denied symptoms such as vomiting, appetite loss, weight loss, or dysmenorrhea. Her past medical history was unremarkable. However, her mother and grandmother had a history of breast carcinoma after the age of 50 years, both treated with surgery followed by adjuvant chemotherapy. Her mother tested negative for BRCA1 and BRCA2 germline mutations.

On general examination, the patient appeared pale, with a body mass index (BMI) of 24.2 kg/m², and her vital signs were stable. Abdominal examination revealed a large, predominantly solid mass in the right iliac fossa, resembling a 20-week gravid uterus. Per speculum examination showed a healthy vagina with a pin-point cervix and bleeding through the external os. Bimanual examination revealed a multiparous uterus deviated to the left, with a large (~12 × 10 cm), predominantly solid lesion originating from the right adnexa, extending anteriorly and superiorly to the uterus and densely adherent to the right lateral uterine border. A freely mobile, purely cystic mass measuring 6 × 5 cm was noted in the left fornix. Her urinary pregnancy test was positive, and her Pap smear was negative for intraepithelial lesions or malignancy.

**Figure 1:**
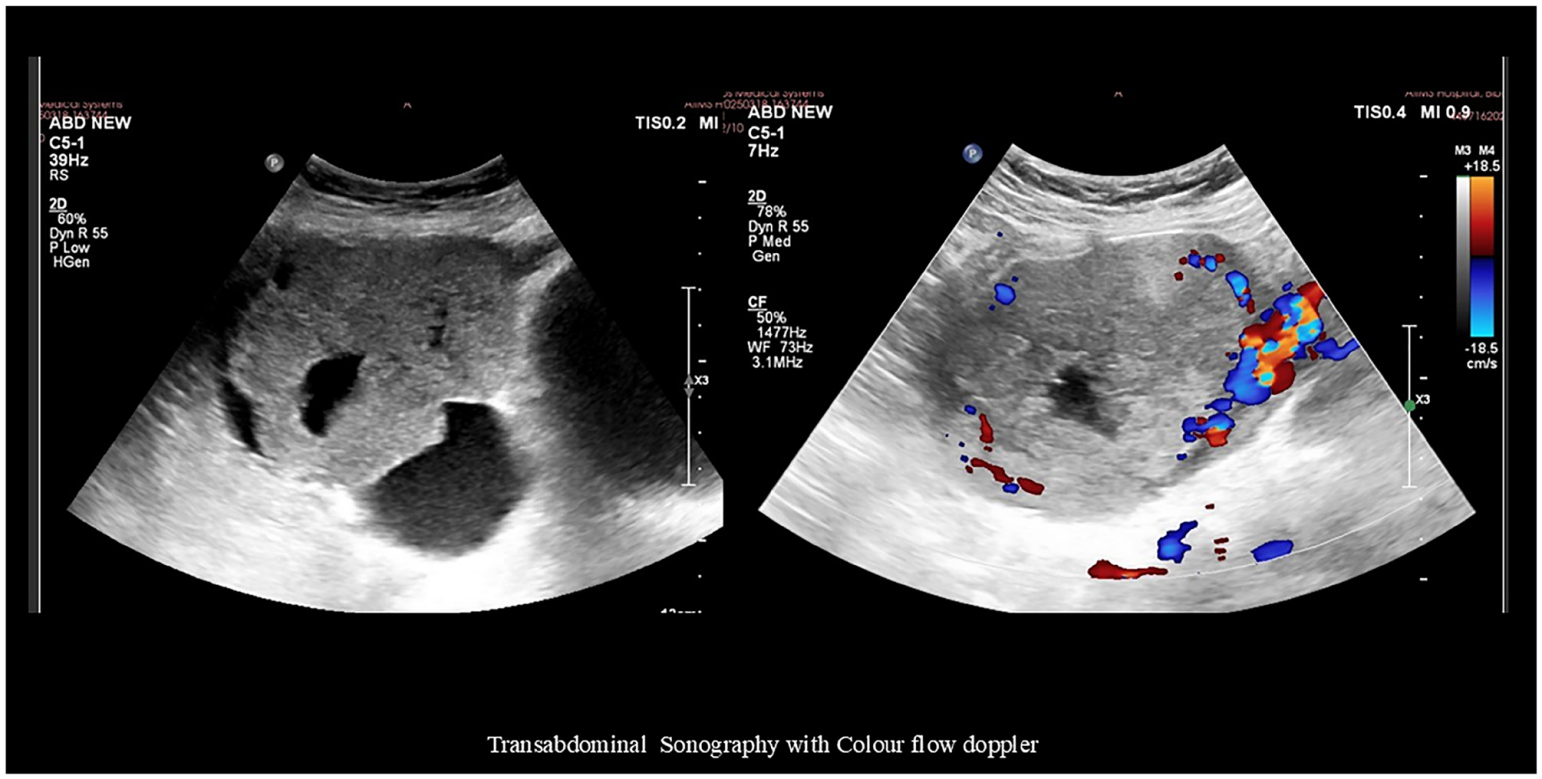
Transabdominal sonography image revealing a well-defined, predominantly solid-cystic lesion (10.2 × 7.8 × 7.8 cm) with vascularized solid components in the right adnexa with areas of hemorrhage.

Given the positive pregnancy test, transabdominal ultrasonography was performed, revealing a uterus measuring 7.7 × 3.4 × 4.8 cm with no gestational sac and an endometrial thickness (ET) of 7 mm. A well-defined, predominantly solid-cystic lesion (10.2 × 7.8 × 7.8 cm) with vascularized solid components was noted in the right adnexa, with areas of hemorrhage suggesting ectopic pregnancy (Figure 1). Another cystic lesion with low-level homogeneous internal echoes, measuring 6.1 × 5.7 × 4.9 cm, was noted in the left adnexa, suggestive of an endometriotic cyst. Laboratory examinations revealed hemoglobin of 8.2 g/dL (normal range: 12–15 g/dL), normal renal (urea: 17–43 mg/dL; creatinine: 0.3–1.3 mg/dL) and liver function tests (Total bilirubin: 0.3–1.2 mg/dL; Aspartate Aminotransferase: 5–35 U/L; Alanine Transaminase: 7–56 U/L; Alkaline phosphatase: 36–104 U/L; Total protein: 6–8.4 g/dL; Albumin: 3.5–5.5 g/dL; Globulin: 2.3–3.7 g/dL), and negative viral markers (HIV, HBsAg, HCV). Tumor markers showed β-hCG: 2,62,809 mIU/mL (normal value: <5 mIU/mL), CA125: 56.5 U/mL (normal value: <35 U/mL), carcinoembryonic antigen (CEA): 1.36 ng/mL (normal range: 0–2.5 ng/mL), CA19-9: 23.6 U/mL (normal value: <37 U/mL), and alpha-fetoprotein (AFP): 6.0 ng/mL (normal range: 0–15 ng/mL), raising suspicion of choriocarcinoma.

**Figure 2:**
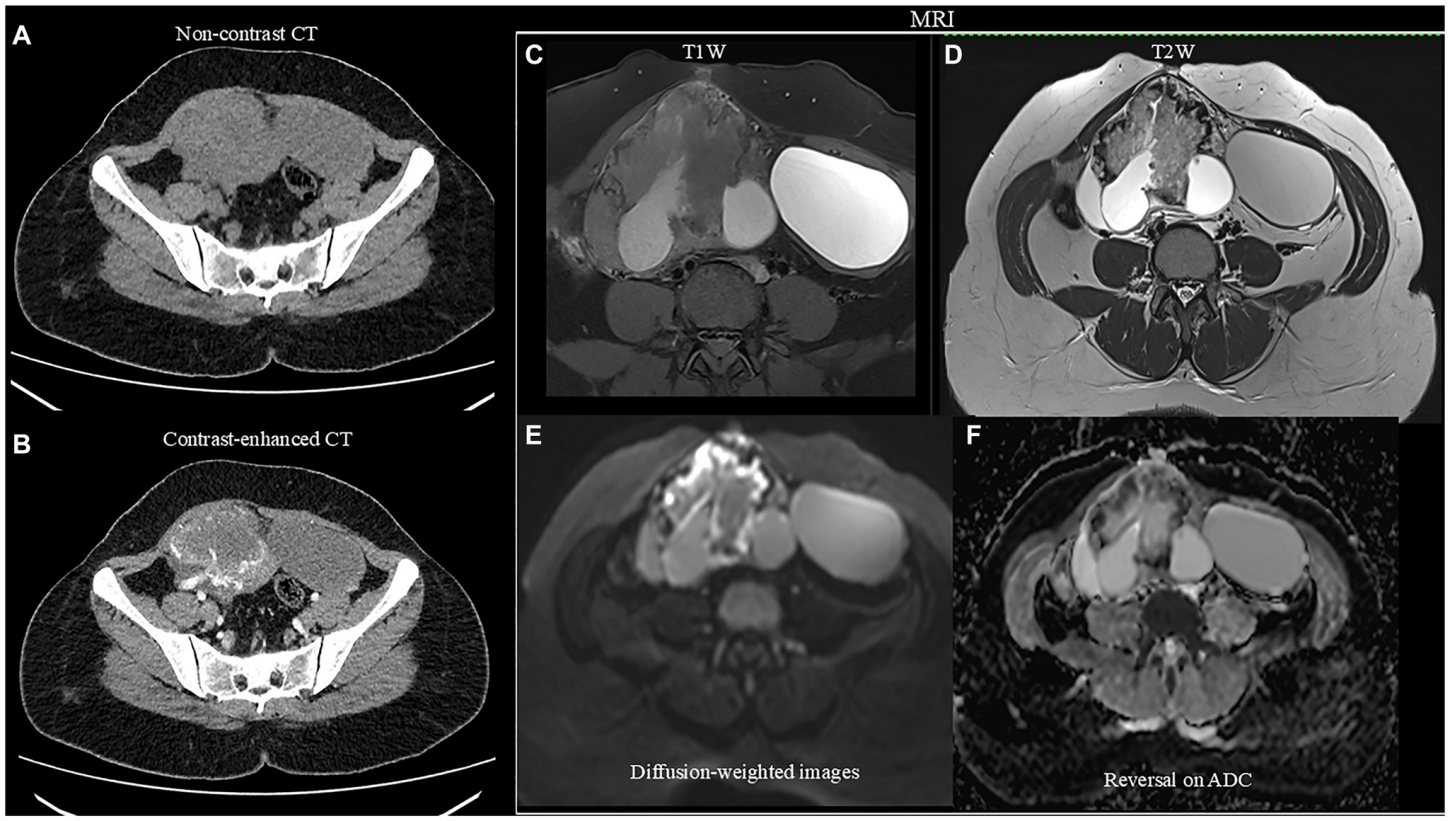
(**A**, **B**) Non-contrast and contrast-enhanced CT images demonstrating a large, solid-cystic lesion arising from the right adnexa. No calcifications were seen on the non-contrast images. Following contrast administration, an intense peripheral enhancement of the solid component was observed, predominantly supplied by a large vascular pedicle originating from the right uterine artery. The right ovary is not visualized separately from the lesion. Additionally, a large cystic structure is noted in the left adnexa; (**C**) Fat-suppressed T1-weighted images revealing a solid-cystic lesion in the right adnexa, with the solid component demonstrating intermediate signal intensity and the cystic areas appearing hyperintense, suggestive of hemorrhagic content. Additionally, a hyperintense cyst was noted arising from the left ovary; (**D**) On T2-weighted images, the solid component of the lesion demonstrates peripheral hypointensity and central intermediate signal intensity, while the cystic component appears hyperintense. The left ovarian cyst shows intermediate signal intensity, suggestive of internal hemorrhage; (**E**) Diffusion-weighted image revealing an undulating peripheral area of diffusion restriction within the solid lesion; (**F**) Showing reversal on Apparent Diffusion Coefficient (ADC), favoring neoplastic etiology.

An MRI scan of the abdomen and pelvis with intravenous contrast was performed to confirm the findings and revealed a uterus measuring 9.1 × 4.2 × 2.3 cm, with an ET of 6.5 mm. The endometrium, junctional zone, and myometrium appeared normal. An ill-defined, mixed solid-cystic lesion (11.8 × 6.8 × 7.3 cm) was seen in the right adnexa extending to the midline pelvis and lower abdomen, with multiple T1, T2, and T2 imaging for iron quantification (TIFS) hyperintense areas suggestive of hemorrhage. A large T1 isointense, T2 heterogeneously hyperintense area with no post-contrast enhancement was observed centrally, surrounded by a thick T2 hypointense area showing blooming on Gradient-Recalled Echo (GRE) sequences. On diffusion-weighted imaging (DWI), an undulating peripheral area of diffusion restriction was seen within the solid lesion. The lesion was closely related posteriorly to the common iliac vessels and abutted the right ureter. Dynamic contrast imaging revealed early and persistent peripheral thick irregular rim enhancement, greater than the enhancement of the myometrium, with no enhancement of solid or cystic components. The right ovary was not seen separately from the lesion. In the left ovary, a well-defined T1, TIFS hyperintense lesion with T2 shading and thin internal septations measuring 4.6 × 7.8 × 7.3 cm was noted (Figure 2C–2F). The MRI findings raised a strong suspicion of an ovarian germ cell tumor, considering the elevated β-hCG levels. CECT of the pelvis, abdomen, lungs, and brain was advised to confirm findings and assess metastasis. CECT revealed a heterogeneous hypodense lesion (11.8 × 7.3 × 6.8 cm) arising from the right adnexa with thick peripheral enhancement and few non-enhancing cystic areas. A non-enhancing cystic lesion from the left ovary (4.6 × 7.4 × 7.8 cm) was also noted (Figure 2A, 2B). No lymphadenopathy or ascites were observed. CECT of the thorax and brain showed no evidence of metastases.

After one unit of blood transfusion, the patient underwent staging laparotomy followed by total abdominal hysterectomy, bilateral salpingo-oophorectomy, and bilateral pelvic lymph node dissection. Intraoperatively, minimal hemorrhagic peritoneal fluid was aspirated for cytology. A large (11 × 10 cm), predominantly solid-cystic, highly vascular mass was found arising from the right ovary, densely adherent to the uterus’s right lateral and cornual ends. The right fallopian tube and ovary were inseparable from the mass. The omentum adhered to the anterior lesion surface with feeding vessels, but no bowel adherence was noted. The uterus was normal-sized. The left ovary harbored a 7 × 5 cm predominantly cystic, freely mobile lesion with the left fallopian tube stretched over its surface. A total abdominal hysterectomy, bilateral masses, and fallopian tubes were removed entirely and sent for histopathology. Infracolic omentectomy and bilateral pelvic lymph node dissection were also performed. No gross tumor deposits were seen over the peritoneum, intestines, liver, or diaphragm undersurface.

**Figure 3:**
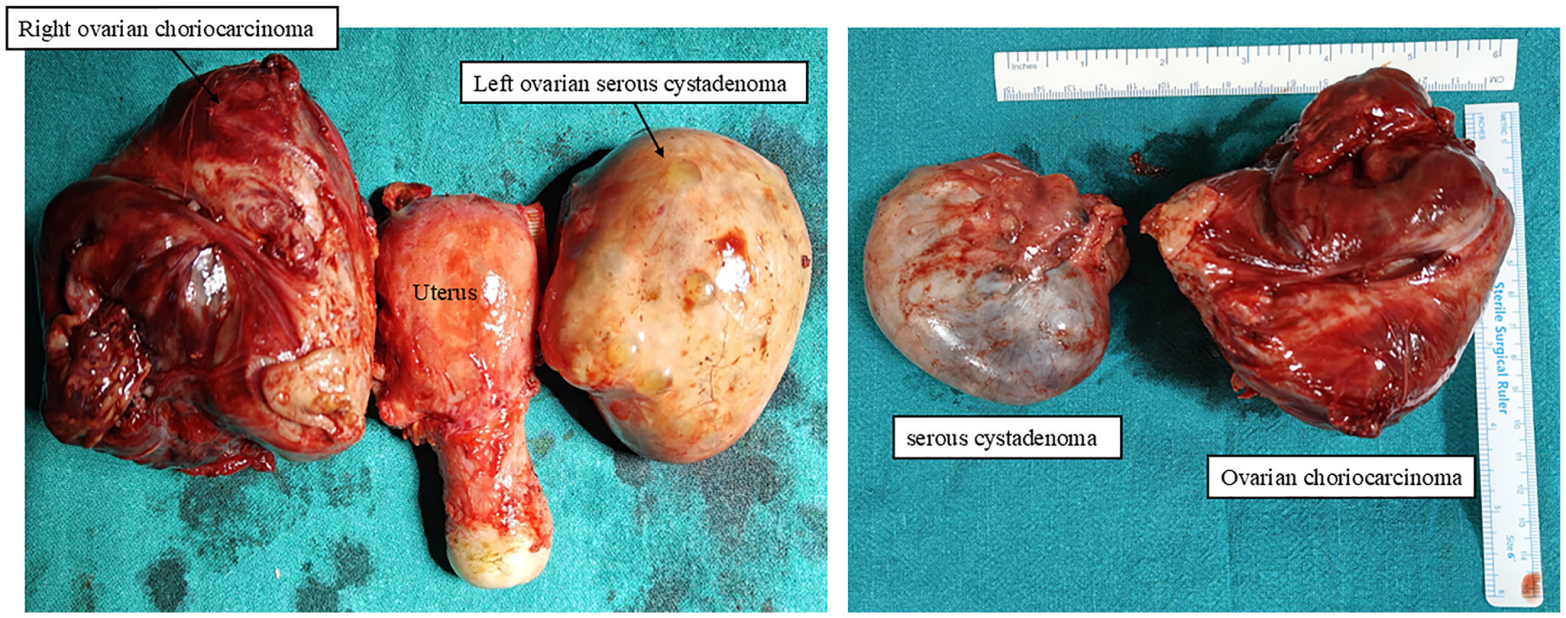
Post-surgery gross images of the uterus with right ovarian choriocarcinoma and left ovarian serous cystadenoma.

Grossly, the right tubo-ovarian mass measured 8.5 × 8 × 7 cm with a lobulated external surface. The cut section showed solid hemorrhagic and necrotic areas measuring 8 × 7.7 × 6.6 cm with no identifiable native tissue. The left tubo-ovarian mass measured 8 × 7 × 3.5 cm, with a smooth external surface; on cut section, a unilocular cyst containing 50 mL hemorrhagic fluid was seen, with no solid areas or papillary excrescences. The left fallopian tube was grossly normal. The uterus measured 6 × 5 × 3 cm with a cervix of 3.5 cm, both grossly unremarkable (Figure 3).

**Figure 4:**
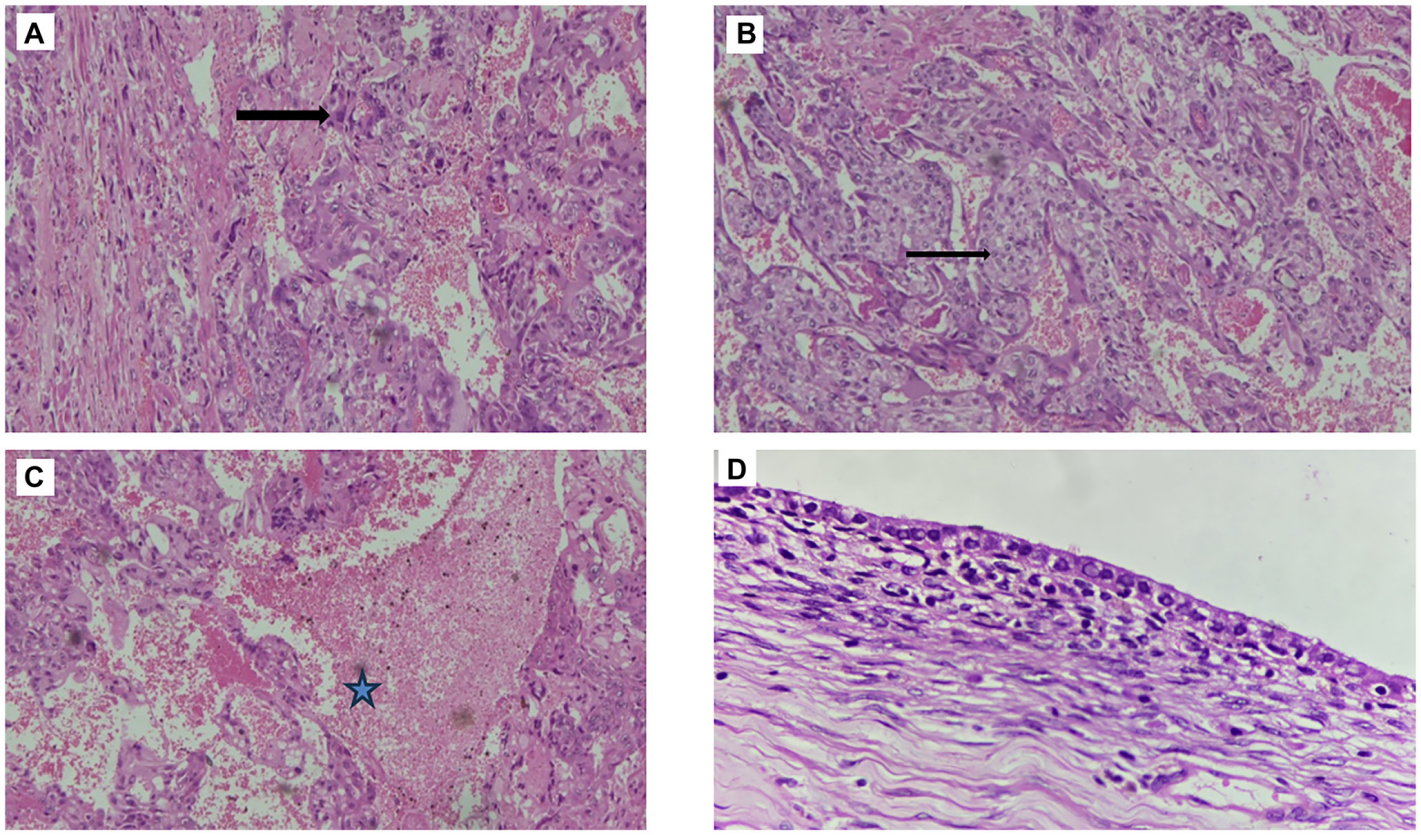
(**A**, **B**) Histopathological images showing tumor cells in the form of mixture of syncytiotrophoblasts and cytotrophoblasts (10X, H&E stain) (arrows); (**C**) Showing tumor cells with hemorrhage and necrosis (10X, H&E stain) (star); (**D**) Left ovarian Serous cystadenoma (H&E, 40X).

Microscopically, the tumor was confined to the right ovary with no normal ovarian tissue identified. No capsular breach was noted. The tumor was composed of cytotrophoblasts and syncytiotrophoblasts with extensive hemorrhage and necrosis; chorionic villi were absent (Figure 4A–4C). The mitotic rate was high (>10 mitoses per high-power field), with atypical mitoses present. No lymphovascular, perineural, or capsular invasion was seen, and surgical margins were tumor-free. Peritoneal fluid cytology was negative for malignancy. The left ovary exhibited serous cystadenoma (Figure 4D), and the endometrium showed disordered proliferative changes. Both the fallopian tubes, peritoneum, omentum, bilateral pelvic lymph nodes, and parametrium were free of tumor deposits. The final diagnosis was stage IA1 right ovarian choriocarcinoma.

**Figure 5:**
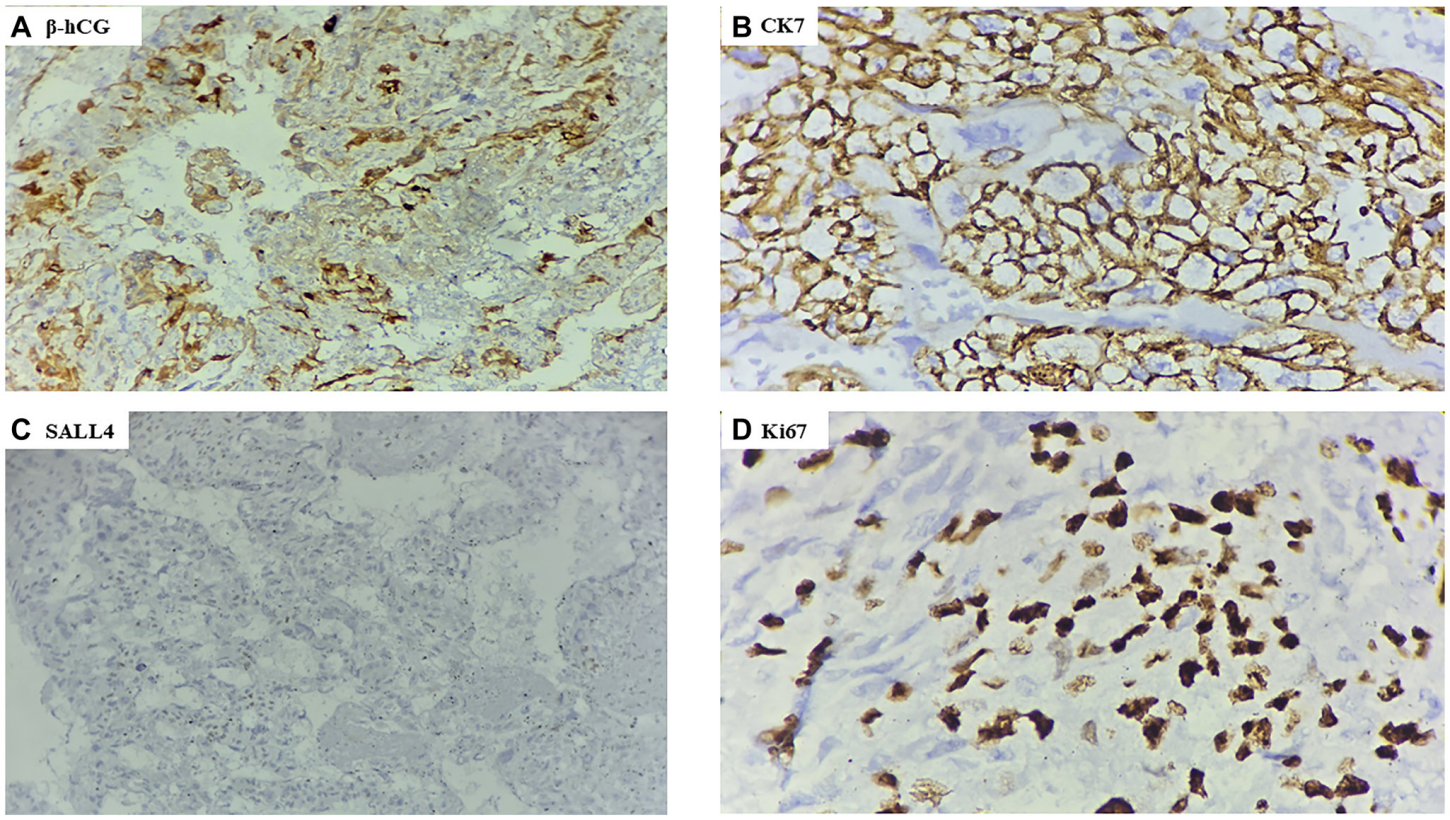
Immunohistochemistry analysis. (**A**) Strong β hCG cytoplasmic positivity in the tumor cells (IHC stain, 40X); (**B**) Cytokeratin (CK) 7 showing membranous positivity in the tumor cells (IHC stain, 40X); (**C**) SALL4 negative in the tumor cells (IHC stain, 40X); (**D**) Ki 67 Ki-67 proliferation index accounting for 70% in the tumor cells (IHC stain, 40X).

IHC confirmed the diagnosis of pure ovarian choriocarcinoma, showing strong positivity for β-hCG and cytokeratin 7 (CK7) and negativity for AFP and SALL4, ruling out other germ cell tumors. The Ki-67 proliferation index was 70% (Figure 5A–5D). To differentiate between gestational and non-gestational ovarian choriocarcinoma, short tandem repeat analysis (a form of polymorphic DNA analysis) was performed, confirming the tumor as pure NGOC.

The postoperative period was uneventful. The patient was discharged in satisfactory condition following stitch removal on the ninth postoperative day. She was advised weekly β-hCG monitoring, which declined from 2,628,095 mIU/mL to <5 mIU/mL after three weeks. She is currently receiving Bleomycin, Etoposide, and Cisplatin (BEP) chemotherapy with bleomycin (15 units), etoposide (100 mg/m²), and cisplatin (20 mg/m²) over five days, in three cycles every 21 days, as the disease was localized. The patient has completed two cycles and remains on regular follow-up with monthly β-hCG testing. Her recent β-hCG levels were <5 mIU/mL, indicating a complete therapeutic response. She is scheduled for monthly follow-up for six months, then three-monthly with β-hCG and Computed Tomography (CT) scans of the abdomen and pelvis, and annually thereafter for two years.

## DISCUSSION

NGOCs are classified into two subtypes: pure and mixed. The pure subtype is exceedingly rare [7], with most cases of primary NGCO occurring in combination with other germ cell tumors, such as teratomas, endodermal sinus tumors, embryonal carcinomas, or dysgerminomas [16]. Pure forms of NGOC arise directly from ovarian germ cells without any association with pregnancy [14]. Its diagnosis is supported by the absence of IHC markers such as Cluster of Differentiation 30 (CD30), Placental Alkaline Phosphatase (PLAP), and AFP, which are typically associated with other germ cell components [17]. Histologically, GOC and NGOC are nearly indistinguishable. Both exhibit marked trophoblastic hyperplasia and anaplasia, lack chorionic villi, demonstrate high proliferative activity, and frequently show intratumoral hemorrhage and necrosis [18]. These overlapping histopathologic features often complicate differentiation between GOC and NGOC, contributing to significant diagnostic challenges [1, 7].

GOC can arise through three primary mechanisms: as a primary tumor originating from an ovarian pregnancy, as a metastasis from a gestational choriocarcinoma located elsewhere in the genital tract, or as a component of a mixed germ cell tumor containing various neoplastic germ cell elements [4]. In contrast, NGOCs are unrelated to pregnancy and typically originate from midline embryonic structures or primordial germ cells within the gonads after birth, exhibiting trophoblastic differentiation [6, 19, 20]. An alternative hypothesis proposes that NGOCs may arise through “retrodifferentiation,” a process in which somatic tumors that have already undergone malignant transformation revert to an earlier, embryonic-like state [1, 21].

Molecular studies have revealed distinct genetic differences between GOC and NGOC. In NGOC, mutations involving *DNAJB9*, a negative regulator of p53, have been identified, along with aberrant p53 expression within tumor cells [22]. NGOC also demonstrates unique copy number variations and significant amplifications of oncogenes such as *HER2*, *IKZF3*, *PGAP3*, and *C-MYC*—genetic alterations not observed in GOC [21]. Additionally, *TP53* mutations have been exclusively associated with NGOC [21]. These genetic changes are thought to contribute to the lower immunogenicity of NGOC, rendering these tumors less responsive to chemotherapy [1].

Histologically, NGOC is characterized by the presence of two distinct trophoblastic cell types: cytotrophoblasts, which form sheets resembling villus-like structures, and syncytiotrophoblasts, which localize at the invasive front of the tumor and are responsible for the secretion of β-hCG and human placental lactogen (hPL) [14]. IHC analysis typically shows tumor cell positivity for β-hCG, hPL, and CK [14].

For diagnosis, pelvic ultrasound is the initial imaging modality of choice. It typically reveals a unilateral—though rarely bilateral—solid, echogenic, and heterogeneous ovarian mass, with a normal-appearing uterus and endometrial thickness [1]. Color Doppler imaging often demonstrates prominent vascularity, characterized by low-resistance arterial waveforms [1, 6]. Further evaluation with CT or MRI is essential for assessing the extent of disease spread and detecting distant metastases [6]. Further evaluation with CT or MRI is essential to assess the extent of disease and identify distant metastases [6]. On CT, NGOC typically present as large, heterogeneous, hypervascular adnexal masses, often demonstrating areas of necrosis and cystic degeneration—features suggestive of malignancy [23]. MRI findings usually reveal well-defined cystic-solid masses, with the solid components showing mixed high and low signal intensities on both T1- and T2-weighted images, along with mildly hyperintense signals on DWI [24].

The staging of NGOC remains unclear due to the limited number of reported cases [3]. Staging is developed using a combination of the 2013 FIGO staging system for ovarian cancer and the 2000 FIGO criteria for choriocarcinoma. Given the aggressive nature of NGOC, early hematogenous and local metastasis is common, with local spread often following the embryologic pathways of germ cell migration [1, 25]. Additionally, NGOCs predominantly metastasize via the lymphatic system [3]. Despite their aggressive behavior and relatively poor overall prognosis, patients with FIGO stage I to III disease have favorable outcomes, with three-year overall survival rates reaching 100% [1]. However, outcomes decline significantly in advanced stages, with FIGO stage IV disease associated with a three-year survival rate of only 25% [1]. Prognosis also varies according to tumor subtype: patients with pure NGOC demonstrate a three-year survival rate of 94%, whereas those with mixed NGOC tumors have a substantially lower survival rate of approximately 50% [1, 10].

Due to the rarity of pure NGOC, no large-scale studies exist to guide optimal surgical management [10, 14]. Available reports indicate that NGOC is typically treated with a combination of surgery and multidrug chemotherapy [9]. Unlike GOCs, which are commonly managed with methotrexate-based chemotherapy guided by the FIGO scoring system—often with single-agent methotrexate for patients with a FIGO score of ≤7 [1, 26] - single-agent chemotherapy is generally ineffective for NGOCs [1]. Given their germ cell origin, NGOCs are treated similarly to malignant germ cell tumors [27] and have a worse prognosis [1, 10].

Platinum-based regimens, particularly BEP, have demonstrated favorable outcomes, with three cycles typically administered for localized disease and four cycles for more advanced or bulky tumors [1, 2, 5]. Radiation therapy is rarely utilized [10]. In young women with suspected stage I disease, fertility-sparing surgery followed by high-dose adjuvant chemotherapy is recommended [1]. Due to the known gonadotoxic effects of chemotherapeutic agents, the co-administration of gonadotropin-releasing hormone (GnRH) analogues has been proposed as a strategy to preserve ovarian function [1, 10].

Treatment response is monitored through serial β-hCG measurements. Following normalization of β-hCG levels, patients require close surveillance: monthly β-hCG tests with chest, abdomen, and pelvis CT scans for the first three months; every three months from months 4 to 12; every six months from months 13 to 36; annually from months 37 to 60; and every two years thereafter [1].

## CONCLUSION

NGOC is a rare, distinct, and highly aggressive tumor that predominantly affects young, reproductive-aged women. It often presents with vague, nonspecific symptoms that mimic ectopic pregnancy or gestational trophoblastic disease and cannot be reliably distinguished from GOC on histopathology alone. Careful evaluation of clinical history to exclude recent pregnancy, along with the use of tissue genotyping when appropriate, is essential for accurate diagnosis. Our case illustrates the classic features of NGOC, including significant bleeding, markedly elevated β-hCG levels, and a unilateral adnexal mass on imaging. Histopathological analysis demonstrated pure NGOC with characteristic cytotrophoblastic and syncytiotrophoblastic elements, with genotypic studies confirming the diagnosis. With timely diagnosis, appropriate chemotherapy, and surgical intervention, patients can achieve favorable outcomes, including prolonged survival and fertility preservation. Nonetheless, NGOC remains a diagnostic and therapeutic challenge, emphasizing the importance of vigilant long-term follow-up and surveillance.
